# An Efficient 24–30 GHz GaN-on-Si Driver Amplifier Using Synthesized Matching Networks

**DOI:** 10.3390/mi14010175

**Published:** 2023-01-10

**Authors:** Lin Peng, Jing Yan, Zhihao Zhang, Gary Zhang

**Affiliations:** 1 School of Information Engineering, Guangdong University of Technology, Guangzhou 510006, China; 2 School of Integrated Circuits, Guangdong University of Technology, Guangzhou 510006, China

**Keywords:** 5G new radio (NR), broadband driver amplifier, GaN HEMT, high efficiency, load-pull, network synthesis

## Abstract

This paper presents a broadband GaN microwave monolithic integrated circuit driver amplifier (MMIC DA) with compact dimensions of 1.65 mm × 0.78 mm for 5G millimeter-wave communication. The optimal impedance domain satisfying the preset goals was first acquired using the simplified load-pull procedure and small-signal simulations, followed by a weighted average method to determine the reference center matching point from which the optimal intrinsic load can be deduced. By means of de-embedding load-pull contours, modeling based on theoretical analysis, and simulation fitting for parameter identification, the nonlinear output capacitance and a series RLC model circuit approximating the input impedance response of the stabilized transistor were extracted. Under the design principle of fully absorbing the parasitic parameters of the device, explicit formulas and tabulated methods related to the Chebyshev impedance transformer were applied to construct filter-based synthesized matching networks at each stage and finally convert them into an implementable mixed-element form via the single-frequency equivalence technique. Measured on-wafer pulsed results for the proposed two-stage DA across 24–30 GHz demonstrated up to 31.1 dBm of saturated output power (P_sat_) with less than 1 dB total fluctuation, 19.3 ± 1 dB of small-signal gain, and 39.8% of peak power-added efficiency (PAE) at the mid-frequency.

## 1. Introduction

During this period of coexistence with the COVID-19 pandemic, online education and cloud offices are on the rise, greatly supported by the extensive 5G wireless infrastructure. Commercial 5G networks have thus far been deployed chiefly in the popular but congested sub-6 GHz frequency range 1 (FR1), with an accelerated evolution toward millimeter-wave (mmW) bands, which possess an abundant spectrum with much wider bandwidths to address the ever-growing demand for mobile data traffic. In particular, the 24–30 GHz range spanning the overlapping bands n257, n258, and n261 that has been planned, licensed, and launched the most by nations worldwide is the focus [1]. Despite their tremendous potential, due to the short wavelengths, mmW communications have some natural drawbacks, such as significant propagation loss and the susceptibility to blockage. Hence, to remedy the lack of mmW signal coverage, the rated output power (P_out_) levels of 5G microcells are often higher. Under such circumstances, a driver amplifier (DA) unit is needed to provide good linearity or efficiency performance as the gain stage prior to the final power amplifier (PA) in an RF transmitter chain [2].

Given that frequency allocations differ by country, a promising low-cost solution from a market perspective is a broadband MMIC DA capable of operating over multiple 3GPP bands in 5G NR FR2, facilitating both the integration and robustness of the front-end system. However, obtaining a sufficient bandwidth, while maintaining decent efficiency in the smallest possible physical footprint poses a challenge to amplifier design because these requirements are mutually restrictive. Leaving aside architectural schemes such as the well-known Doherty and outphasing amplifiers, which are mainly intended to improve the *average* efficiency within a limited bandwidth and also necessitate substantial components, a regular single-way structure would be more attractive in terms of implementation complexity, for which, the primary efficiency enhancement technique is the so-called waveform engineering. Conventional harmonic-tuned class-F/F^−1^ PAs strive to create a specific impedance environment that shapes output V–I waveforms with less crossover for greater *absolute* efficiency but a narrow fractional bandwidth (FBW) [3]. Continuous class-J PAs, proposed later, subtly mitigate this shortcoming through the “design space” concept, which alleviates stringent harmonic impedance requirements, reaching the same high efficiency as those in the normal class-B mode by only manipulating up to the second harmonic load [4,5]. These amplifiers, however, are typically biased at a class-B or deep class-AB condition and added with harmonic control circuitry, resulting in a poor linear drive capability and higher area consumption. Therefore, we need to focus on the broadband aspect to find a satisfactory solution. More specifically, we should look for appropriate matching approaches to present the optimal complex load and source impedances to the device within the designated broadband. The simplified real-frequency technique (SRFT) directly processes gathered discrete load data with computer-aided design (CAD) programs, solving least-squares problems to build broadband matching networks (MNs) without modeling the load or relying on expertise to predefine a proper equalizer topology [6]. However, when the classical Levenberg–Marquardt (LM) algorithm is applied to optimize the transducer power gain function, the convergence speed and results are sensitive to the initial assignment of multiple independent variables; substituting with a more robust genetic or hybrid algorithm can help search for a global optimal solution [7,8], but at the price of increased computational complexity, which limits the practicality of the SRFT. Another category of analytical methodology, based on filter synthesis, yields closed-form solutions for simple loads of RC, RL, and RLC [9,10,11], which is mature in theory and easier to implement than CAD-dependent numerical optimization. MNs with different passband properties can be configured according to the load composition, the mainstream being Butterworth, Chebyshev, and Elliptic types and their derivatives [11,12,13,14,15,16]. The filter-matching method is flexible enough to absorb parasitic parameters at both the input and the output of the transistor, enabling broadband impedance transformation and strengthening amplifier band selectivity, while reducing harmonic energy loss.

Last but not the least, in MMIC performance, device technology is the bottleneck because the radical way to extend the bandwidth is to reduce the MN’s impedance transformation ratio (ITR), and attaining the requisite amount of forward gain with fewer amplification stages effectively promotes efficiency. Compared to the widespread Si- and GaAs-based semiconductors, emerging GaN high-electron-mobility transistors (HEMTs) have a large material bandgap (3.4 eV) and excellent thermal tolerance, allowing them to run at elevated drain voltages, power densities, and ambient temperatures and thus support smaller form factors. Moreover, they feature a high electron saturation velocity and port resistances with low parasitic capacitances, making them ideal for broadband high-efficiency amplifier designs in the mmW regime [17,18,19,20].

The main scope of this paper is to present a comprehensive procedure of a small-scale 1-watt GaN-on-Si DA covering the range of 24–30 GHz, which is rarely reported in most of the published GaN MMIC literature. On the basis of the optimal impedance domain determined by simplified load-pull and small-signal simulations, together with equivalent input and output impedance models of the stabilized device, the filter synthesis theory was applied to complete the design of MNs so that power cells could perform well. The experimental results of the proposed DA are summarized and compared to those from recently published studies.

## 2. Circuit Design Considerations

**Figure 1:**
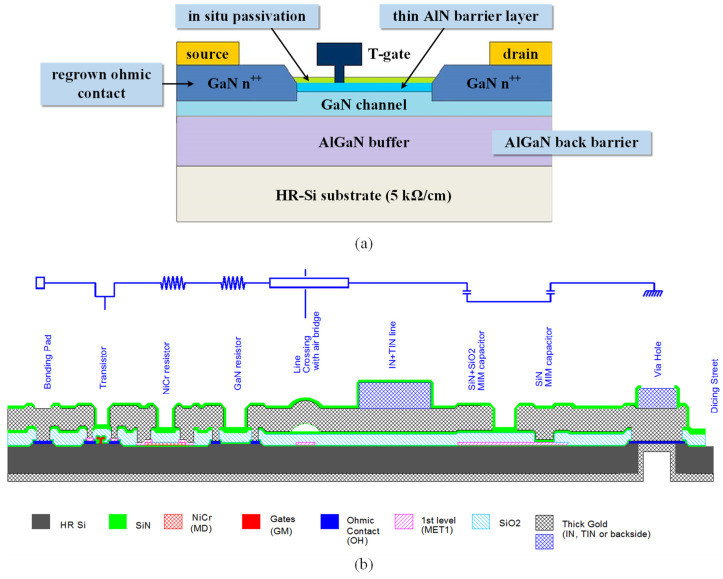
(**a**) Epitaxial structure and (**b**) cross-section of OMMIC’s 0.1 μm GaN-on-Si process.

The adopted technology is OMMIC’s (OMMIC SAS, Limeil-Brévannes, France) 0.1 μm double-heterojunction AlN/GaN/AlGaN HEMT process known as D01GH, based on a 3-inch high-resistivity silicon substrate thinned down to 100 μm with a dielectric constant ε_r_ of 11.7 and a loss tangent tg(δ) of 0.015. As depicted in Figure 1, it uses in situ passivation to avoid memory effects and regrown non-alloyed ohmic contacts to minimize access resistance. The thin AlN barrier serves to diminish short-channel effects, whereas the AlGaN back barrier improves electron confinement. Mushroom gates of 100-nm-length and a short gate–source distance enable the cutoff frequency (*f_T_*, i.e., *f* @ |H_21_| = 1) to exceed 100 GHz. The typical RF power density is around 3.3 W/mm @ 30 GHz but can go up to 5.7 W/mm for a recommended bias supply *V_D_* of 12 V, with a gate–drain breakdown voltage of 36 V. Depletion-mode transistors have been fully modeled, taking into account the electro-thermal nonlinear attribute. Various passive components, including spiral inductors, NiCr thin-film resistors (40 Ω/□), GaN resistors (400 Ω/□), and two types of MIM capacitors (50 pF/mm^2^ and 400 pF/mm^2^), via holes and microstrip lines with metal thickness options of 1.25 μm or 2.5 μm, are available in the foundry design kit [21].

The process we chose is built on a Si platform rather than the general silicon carbide (SiC) one, which has superior thermal conductivity along with low loss. On the one hand, the required power levels for 5G mmW applications make the adoption of a Si substrate feasible, and the other major reason is that SiC remains expensive and subject to export controls, whereas Si-based GaN is relatively cheap, mature enough, and compatible with heterogeneous integration with SiGe/CMOS [22]. It offers a competitive performance and the potential for large economies of scale, opening up the possibility of a realistic volume production of 5G MMICs.

### 2.1. Fundamental Load-Pull Analysis and Determination of the Optimal Impedance Domain

In consideration of the maximum available gain of transistors, estimated transmission link loss, process variation, and design redundancy, a cascaded two-stage common-source architecture was employed for the DA. Transistor peripheries were staged at a ratio of 1:2, with a 46 × 8 μm cell chosen for the latter stage, to meet the specifications of 1 W P_sat_ with over 30% PAE and beyond 18 dB of linear gain. Due to the nonlinear nature of the device and essentially the output parasitic capacitance *C_out_*, which strongly depends on the operating conditions and transistor geometry, the trajectory formed by connecting the optimal load impedance points *Z_L,opt_* obtained through load-pull incrementally by frequency travels counterclockwise when mapped on the Smith chart. However, the recorded impedance locus *Z_L_(f)* after transforming the standard 50 Ω by the output matching network (OMN) rotates in the reverse direction with increasing frequency compared to the former. Consequently, even if these two profiles happen to be exactly congruent, phase mismatch will exist for all but one frequency. This unfavorable phenomenon of opposite impedance rotation explains why the amplifier is prone to severe mismatch at and near the edge frequencies of the wide target band, leading to a dramatic deterioration in performance, thus troubling the design of the broadband MN. Instead of attempting to track the frequency-dependent *Z_L,opt_* over broadband at the expense of a high component overhead, as in the common strategy, we defined an optimal impedance domain that fulfills the preset goals of PAE > 40%, P_1dB_ > 31 dBm, and output-stage gain > 8 dB. Meanwhile, a compromised center impedance *Z_L,ctr_* was identified. The DA’s broadband output capability is basically assured as long as the in-band *Z_L_(f)* rotates in a knotted shape around *Z_L,ctr_* and falls inside the prescribed constraint space. In addition, considering that the transistor’s gain roll-off characteristics and the actual bandwidth may narrow or frequency-shift from evaluation, the circuit-level design should prioritize an upper-frequency performance, while introducing some low-frequency mismatch to enhance the broadband effect via stagger-tuning for a balanced large-signal response [17].

In mmW bands, *C_out_* shorts out most of the harmonic contents. Therefore, sophisticated harmonic control is no longer viable. As harmonics are far from the passband that can be readily suppressed by a low-pass OMN, the impacts of harmonic loads were first ignored in the course of successive single-tone load-pull simulations by initializing them and source harmonic terminations as open-circuit (e.g., 1 kΩ). It is worth mentioning that a simplified alternative was used here, unlike the conventional process, where the load-pull needs to be iterated with the source-pull to get optimal convergence results. We regarded the HEMT with a parallel RC stabilization network as a new cell, figuring out the voltage-to-current ratio at the equivalent gate node when the cell is excited to the P_1dB_ state by a continuous-wave (CW) signal to derive the fundamental input impedance *Z_in,fund_* and then took its conjugate as the source impedance *Z_S,fund_*. Since the device’s non-unilateral operation and PAE is more critical, *Z_S,fund_* must be updated with the variation of *Z_L,opt_* picked at the best PAE for each iteration until the two, which interact, cease to change, i.e., each converges to a certain fixed value. Table 1 compares the conventional and simplified load-pull scripts conducted on the last stage at 24 GHz, 28 GHz, and the extended corner of 32 GHz under the same simulation conditions. There is only a negligible difference in the PAE between them, and the corresponding *Z_L,opt_* are identical, so the source-pull step for seeking the optimal source impedance *Z_S,opt_* can be omitted to facilitate a more in-depth load analysis with guaranteed correctness.

**Figure 2:**
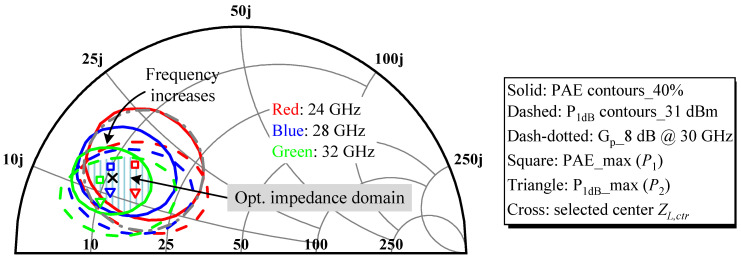
Simulated load-pull contours at 1 dB gain compression and the G_P_ circle of 8 dB at 30 GHz for the output stage cell when given a nominal bias point of *V_D_* = 12 V and *V_G_* = –1 V, which yields the maximum device DC and AC transconductance [17].

With this simplified method, joint small-signal simulations for generating constant power gain (G_P_) circles resulting from load mismatch allow the rapid determination of the wanted optimal impedance domain, shaded in Figure 2. About 2/3 of its boundaries are bound by the contours of PAE and P_1dB_ at 32 GHz due to intensifying parasitic effects (mainly *C_out_*) that cause the contours to move counterclockwise toward the real axis of the Smith chart and gradually shrink as the frequency increases. In addition, the design margin of 2 GHz extended to higher frequencies is an experience-based trade-off and the concentric circle distribution of G_p_ outcomes in Figure 2 is drawn only for the inner circle at 30 GHz, which is just externally tangent to the optimal domain where three groups of contours overlap. The best performance marks *P*_1_ and *P*_2_, wrapped around the PAE and P_1dB_ contours, respectively, do not coincide, and they each become closer at higher fundamentals, which in turn are farther apart from one another at the lower side. To accommodate the uneven dispersion of contour peaks, a two-round weighted average calculation was proposed to identify the suitable *Z_L,ctr_*. In the beginning, a weighted interpolation was carried out between *P*_1_ and *P*_2_ for every designated frequency. Because PAE is the primary concern, a weight of 2/3 was assigned to *P*_1_ to bring the interpolation point *P_i_* closer to *P*_1_. The first round of applying Equation (1) yields *P_i_* at three frequencies, listed in Table 2. On top of that, recognizing the importance of medium- and high-frequency performance, similarly *P_i_* at 24, 28, and 32 GHz was assigned the weighting factors *W_i_* of 0.2, 0.5, and 0.3, respectively, in accordance with Equation (2) to obtain the *Z_L,ctr_* trade-off efficiency and P_out_ of 11 + j12.9 Ω.

(1) $$\begin{array}{ll} P_{1} & =x_{1}+jy_{1}\\ P_{2} & =x_{2}+jy_{2}\\ P_{i} & =x_{i}+jy_{i}=\frac{2}{3}P_{1}+\frac{1}{3}P_{2}\\ & =\frac{2}{3}x_{1}+\frac{1}{3}x_{2}+j\left(\frac{2}{3}y_{1}+\frac{1}{3}y_{2}\right) \end{array}$$

(2) $$Z_{L,ctr}=\sum P_{i}W_{i}=\sum x_{i}W_{i}+j\sum y_{i}W_{i}$$

**Figure 3:**
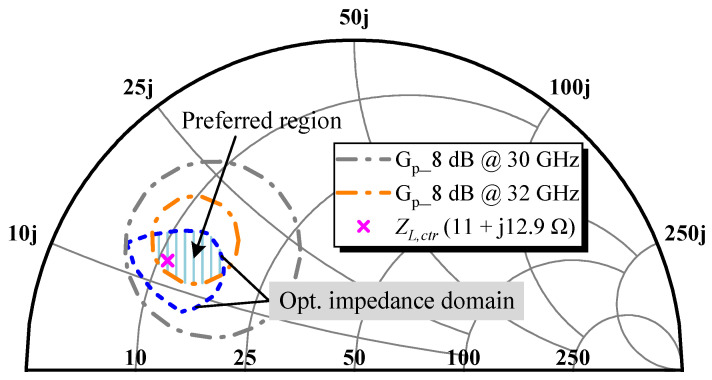
Preferred region in the optimal impedance domain.

For more precise guidance on the OMN design and to consolidate the comprehensive performance of the DA, a G_p_ circle of 8 dB at 32 GHz has been added in Figure 3, whose intersection with the acquired optimal impedance domain represents the desired matching zone under stricter conditions. It contains *Z_L,ctr_*, but the range is cut in half from the previous one, which will probably increase the matching difficulty. As G_p_ is a less prominent metric, the newly planned impedance space was handled as a preferred region rather than a mandatory objective.

**Figure 4:**
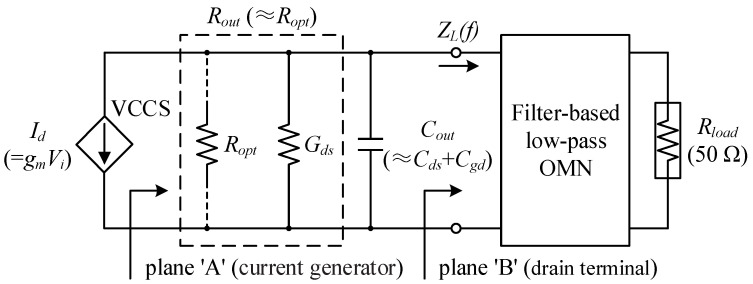
Equivalent parallel RC model for the large-signal output impedance of an HEMT.

In any case, it is necessary to establish the cell’s impedance model to analyze broadband matching and as a meaningful reference for deciding the network topology. Because the intrinsic output circuit of a field-effect transistor (FET) could be thought of as a parallel connection of the voltage-controlled current source (VCCS), internal conductance *G_ds_*, and *C_out_*, the output impedance of a GaN HEMT equates to the parallel *R_out_C_out_* model, as illustrated in Figure 4. *C_out_* is mostly made up of *C_ds_* and *C_gd_*, with *C_gd_* playing a minor role. The optimal load resistance *R_opt_* can be roughly estimated using the Cripps loadline method [4], written in Equation (3); empirically by Equation (4); or straight from the acquired *Z_L,ctr_*, as indicated in Equation (5). These correspond to *R_opt_* values of 40 Ω, 32 Ω, and 26 Ω for the adopted 46 × 8 μm device, with *V_D_* = 12 V, knee voltage *V_knee_* = 2 V, maximum current *I*_max_ = 0.5 A, and conservative simulation target *P_out_* = 32 dBm. Since the classic loadline theory is based on several ideal assumptions and does not account for non-negligible parasitic effects regardless of operational class, the *R_opt_* inverse from load-pull results will be more in line with the practical scenario. *C_out_* can be obtained in a similar manner. However, note that unlike *R_opt_*, which is somewhat customized, *C_out_* is an innate parameter of the device, with an exact value in a given condition. Thus, there will be some errors when using the familiar extraction Formula (6), but the computed figure of 0.26 pF at 28 GHz is worth taking as an initial guess for *C_out_*. Next, different *C_out_* values are de-embedded from the load-pull contours derived at plane ‘B’ (see Figure 4) in 0.01 pF steps within a small interval of 0.24–0.28 pF. When their conjugate mirror contours are observed back to the position symmetrical to the real axis of the Smith chart, the de-embedding process is judged as complete, as contours at the current generator plane ought to be frequency-independent [4,23]. *C_out_* was then identified to be 0.27 pF. In the same way, the *C_out_* of the 46 × 4 μm cell is 0.13 pF, and the *R_opt_* for the driver stage was selected as 75 Ω.

(3) $$R_{opt}=\frac{2\left(V_{D}-V_{knee}\right)}{I_{\max}}$$

(4) $$R_{opt}=\frac{{\left(V_{D}-V_{knee}\right)}^{2}}{2P_{out}}$$

(5) Ropt=1ReYL,ctr*YL,ctr*=1conjZL,ctr

(6) Cout=ImYL,ctr*ω

### 2.2. Harmonic Load-Pull Analysis and Determination of the Phase Avoidance Interval

**Figure 5:**
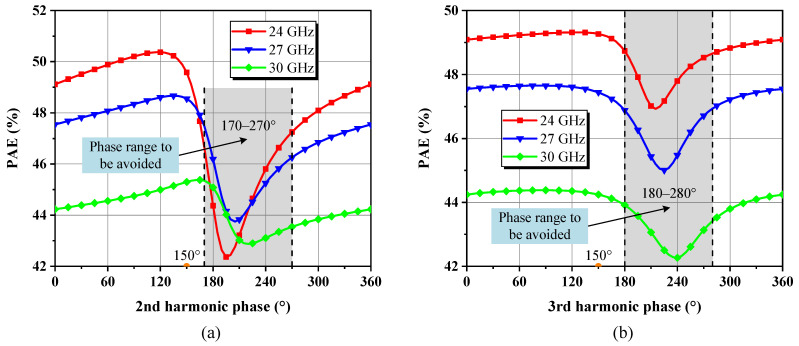
Influence of the (**a**) second and (**b**) third harmonic phases on simulated PAE at different frequencies for a constant 0.95 magnitude of load reflection coefficient when given 23 dBm of available *P_in_*.

**Figure 6:**
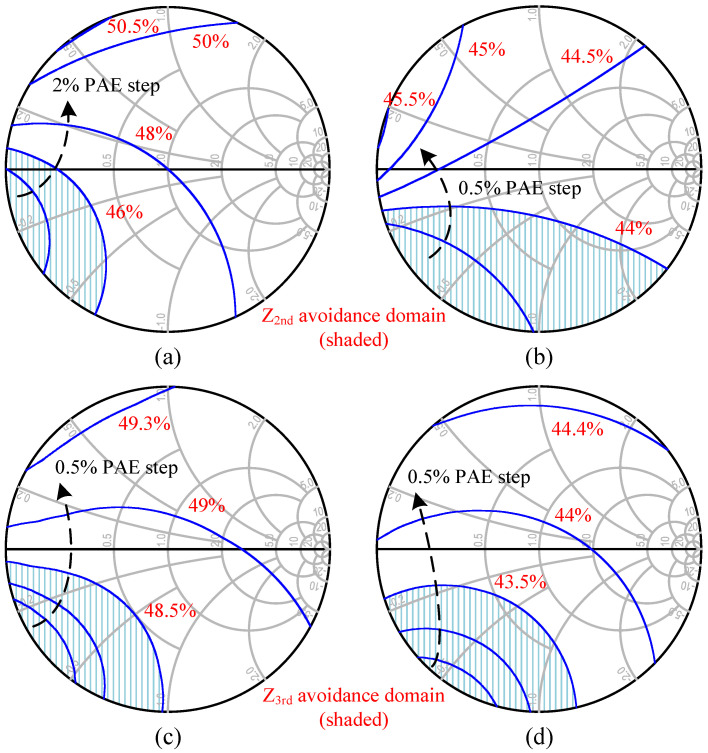
Harmonic load-pull simulation results: second harmonic at (**a**) 24 GHz and (**b**) 30 GHz; third harmonic at (**c**) 24 GHz and (**d**) 30 GHz.

According to energy conservation law, the total source power comprising the DC supply and the incident power *P_in_* amounts to the *P_out_* at fundamental and harmonic frequencies, plus the power dissipates into heat in the transistor, written as Equation (7). Harmonic impedances could be treated as almost purely reactive, i.e., the harmonic power contribution is modest, but they still act on the overlap of output V–I waveforms in the time domain, altering *P_diss_*. Thus, the second and third harmonic loads were varied one by one along the near periphery of the Smith chart, while keeping *Z_L,ctr_* at all fundamentals and the open circuit for other harmonic terminations to investigate the effect of their phase on the PAE of the 46 × 8 μm cell separately [14,24]. As seen from Figure 5a, the resulting PAE drops remarkably once the optimal phase is reached and curve families show deep notches in the 170–270° range, which indicates that the second harmonic termination leads to an increase in unwanted power dissipation when it is transformed into this phase interval. The peak-to-peak PAE reduction is about 8% to 3% from 24 GHz to 30 GHz. In contrast, except for a milder decline in the range of 180–280°, PAE curves in Figure 5b fluctuate little in the rest of the phase interval, which can be accepted as suitable ranges. These findings echo the earlier supposition that higher-order harmonics dampened by *C_out_* lack enough strength to significantly affect the drain voltage waveform and thus are less effective against PAE. For most cases, only the first two-order harmonics deserve to be discussed. Manipulating more but minor harmonic objects in the broadband will multiply layout patterns and introduce higher losses than they are worth. Additionally, after repeating the present simulations at different gain compression points corresponding to *P_in_*, we found that the phase ranges to be avoided converge, just with distinguishable differences in the degree of depression, and the deviation of the phase of the load reflection coefficient at the PAE valley due to *P_in_* variations is less than 10° for each frequency. Figure 6 shows the detailed harmonic load-pull simulation results for PAE at 23 dBm of *P_in_*.

(7) $$P_{DC}+P_{in}=P_{out}+P_{diss}=P_{out,f}+\sum_{n=2}^{\infty }P_{out,nf}+\int_{T}v_{DS}\left(t\right)\cdot i_{D}\left(t\right)dt$$

In summary, the OMN design should strike a balance to prevent the harmonic impedances, especially the second one, from falling into the low-efficiency region. Fortunately, the wide tolerance range of 250° allows us to confidently concentrate on developing the fundamental MN, supplementing interventions with harmonic control, if necessary. The preferred phase location is considered between 0 and 150°.

## 3. Design and Implementation of Matching Networks

### 3.1. Mixed-Element Realization Method and Layout Considerations

Objectively speaking, in a manufactured MMIC, only some operable parameters of components can undergo limited unidirectional adjustment by physical trimming (e.g., laser), which requires sensible forethought and a well-planned setup during the design phase. The difficult-to-change nature implies that to accurately anticipate the real-world behavior of MMICs, especially for compact designs, we need rigorous electromagnetic (EM) simulations, which are time-consuming. When individually designed MNs and cells are cascaded into a complete amplifier following a generic modular implementation process, significant performance offsets tend to occur which are hard to eliminate. Each modification often involves performing thorough EM/circuit co-simulations to understand the corresponding knock-on effects, making it difficult to locate the root causes of problems or sensitive factors.

In view of the above facts, the schematic design alternated with layout replacement, continuously considering various EM influences throughout the development procedure, and the overall circuit was built in a step-by-step fashion to greatly reduce the difficulty and the number of optimization iterations in the final joint-tuning phase, ensuring that the desired results can be obtained efficiently.

**Figure 7:**
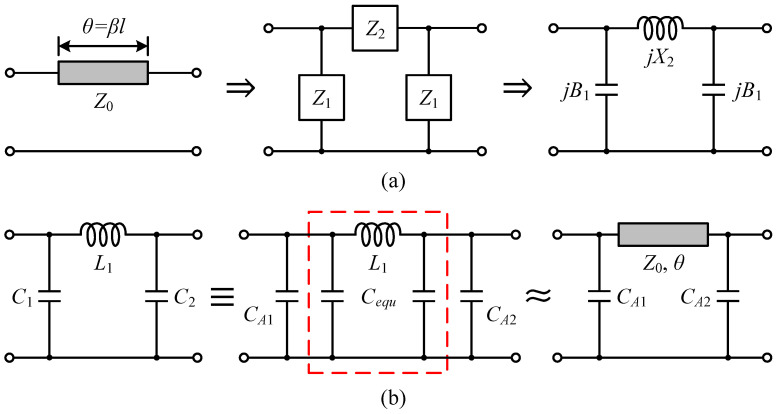
(**a**) Equivalent two-port network of a transmission line. (**b**) Swap an identified lumped low-pass π-section into its almost equivalent mixed-element counterpart using the replacement technique.

For layout convenience and physical feasibility, MNs were constructed in a mixed-element style with microstrip lines and MIM capacitors. Generally, a section of transmission line with electrical length *θ* and characteristic impedance *Z*_0_ can be represented by a symmetric π-shaped network, described in Figure 7a. According to the definition of the Z-matrix, we have

(8) $${\left.Z\right|}_{Tline}=\left[\begin{array}{cc} -jZ_{0}\cot\theta & -jZ_{0}\csc\theta \\ -jZ_{0}\csc\theta & -jZ_{0}\cot\theta \end{array}\right]$$

(9) $${\left.Z\right|}_{\pi }=\left[\begin{array}{cc} \frac{Z_{1}\left(Z_{1}+Z_{2}\right)}{2Z_{1}+Z_{2}} & \frac{Z_{1}^{2}}{2Z_{1}+Z_{2}}\\ \frac{Z_{1}^{2}}{2Z_{1}+Z_{2}} & \frac{Z_{1}\left(Z_{1}+Z_{2}\right)}{2Z_{1}+Z_{2}} \end{array}\right]$$

Letting *Z*|*_Tline_* = *Z*|_π_, gives

(10) $$Z_{1}=\frac{Z_{0}}{j\tan\left(\theta /2\right)}=\frac{1}{jB_{1}}$$

(11) $$Z_{2}=jZ_{0}\sin\theta =jX_{2}$$

For a transmission line segment whose physical length *l* is much smaller than the wavelength *λ* (*l* < *λ*/8), its equivalent parallel susceptance and series reactance are proximately linear to *ω*, that is

(12) $$B_{1}=\frac{\tan\left(\theta /2\right)}{Z_{0}}\overset{\theta <45{}^{\circ}}{\approx }\frac{\beta l}{2Z_{0}}=\omega \frac{l}{2v_{p}Z_{0}}=\omega C_{equ}$$

(13) $$X_{2}=Z_{0}\sin\theta \overset{\theta <45{}^{\circ}}{\approx }Z_{0}\beta l=\omega \frac{Z_{0}l}{v_{p}}=\omega L_{equ}$$

where *v_p_* is the phase velocity and *β* is the propagation constant. Therefore, the single-frequency equivalence technique illustrated in Figure 7b enables the approximate substitution of a series inductor with a distributed element [25]. Firstly, the obtained lumped prototype is decomposed into several cascaded subsections as needed, and one of the lossless units *C*_1_-*L*_1_-*C*_2_ is designated. Then, a prescribed symmetric π ladder with parallel capacitors *C_equ_* centered on the inductor *L*_1_ is abstracted from its interior and replaced by a commensurate transmission line using the relations shown in Equations (12) and (13), with the chosen *Z*_0_ and in-band angular frequency *ω*_0_, where *L_equ_* = *L*_1_, *ω* = *ω*_0_, and the *C*_1_ and *C*_2_ are deemed to be no less than *C_equ_*. The termination capacitors *C_An_* in the resulting mixed-element network are

(14) $$C_{An}=C_{n}-C_{equ}=\frac{\cos\theta +\omega_{0}^{2}L_{1}C_{n}-1}{\omega_{0}^{2}L_{1}}\;\left(n=1,2\right)$$

In cases where the complete *C*_1_-*L*_1_-*C*_2_ combination cannot be divided, Equation (12) suggests that the remaining fringe capacitance *C_equ_* can be reduced by selecting a larger *Z*_0_, hence decreasing the perturbation caused by unequal substitution on matching. Further, the narrow transmission line facilitates repeated bends to save the layout area, and as reflected by Equation (13), it also behaves more like an inductor. Thereby, small transmission line widths were chosen here. The line width was set to 10 μm with the exception of the OMN, where the line width was set to 15 μm. Supply routes and the OMN were deliberately thickened with double metal to diminish ohmic losses, enhancing the current handling capability for the former. In addition, 45° segments with perpendicular access lines were used at the corners to mitigate discontinuities and a gap of at least three times the line width was maintained between the adjacent matching microstrip lines to lower signal coupling. The 2.5D field simulator (Momentum) built into the Advanced Design System (ADS) software was used to solve all kinds of EM effects.

### 3.2. Synthesized Low-Pass OMN and ISMN

The Bode–Fano criterion clarifies the relationship between bandwidth Δ*ω* and the reflection coefficient Γ(*ω*). For the parallel RC-type load, the achievable lossless MN must comply with the constraint (Equation (15)), where Γ*_m_* is the minimum return loss assumed to be constant over a certain frequency bandwidth ∆*f*. *R_opt_* and *C_out_* are now known to be 26 Ω and 0.27 pF, respectively, and with an ideal *S*_11_ of –25 dB, the theoretical limit ∆*f* can be calculated from the rearranged Equation (16) as 24.75 GHz, which is four times the actual demand. As a result, a large enough target simulation bandwidth *BW_sim_* could be chosen for the OMN, while maintaining a low passband ripple, yet this implies additional cost and insertion loss simultaneously. Bearing in mind the principle of miniaturization and design margin, the *BW_sim_* was set to be 22 to 32 GHz, a compromise, and the rest of the MN design would follow suit.

(15) $$\int_{0}^{\infty }\ln\frac{d\omega }{\left|\Gamma \left(\omega \right)\right|}=\Delta \omega \ln\frac{1}{\Gamma_{m}}\leq \frac{\pi }{R_{out}C_{out}}\approx \frac{\pi }{\left(\frac{R_{opt}}{1+R_{opt}G_{ds}}\right)\left(C_{ds}+C_{gd}\right)}\approx \frac{\pi }{R_{opt}C_{out}}$$

(16) $$\Delta f=\frac{1}{2R_{opt}C_{out}}\ln^{-1}\frac{1}{10^{S_{11}\left(dB\right)/20}}$$

It is possible to have infinite ∆*f* with zero value capacitance, so properly handling *C_out_* is the key to realizing a broadband OMN. There are two main ways: (1) tune existing matching elements or add specialized susceptance cancelation circuitry to minimize the effect of *C_out_* [26,27], where passive negative susceptance networks, such as a compensating shunt inductor are preferable and more prevalent [23] and (2) absorb *C_out_* into matching. Unfortunately, the *C_out_* of 0.27 pF seems so large that neither compensation nor absorption appears to be a cost-effective or even a feasible option for on-chip wideband matching. To think otherwise, since *Z_L,ctr_* is the trade-off matching point for the entire target band, and not far from the real axis of the Smith chart, the OMN design can start with an impedance transformation from 48 Ω to real(*Z_L,ctr_*) and imag(*Z_L,ctr_*) is regarded as another sense of the quantity to be compensated. It should be noted that the L-shaped MN consisting of the parasitic shunt capacitor of the 100 μm square output pad and a 1.8 pF DC-block capacitor shifts the external 50 Ω load slightly down to the capacitive half-plane of the Smith chart, which was pulled back to 48 Ω at 27 GHz in advance by a high-impedance microstrip line. The RF input side received the same treatment.

**Figure 8:**
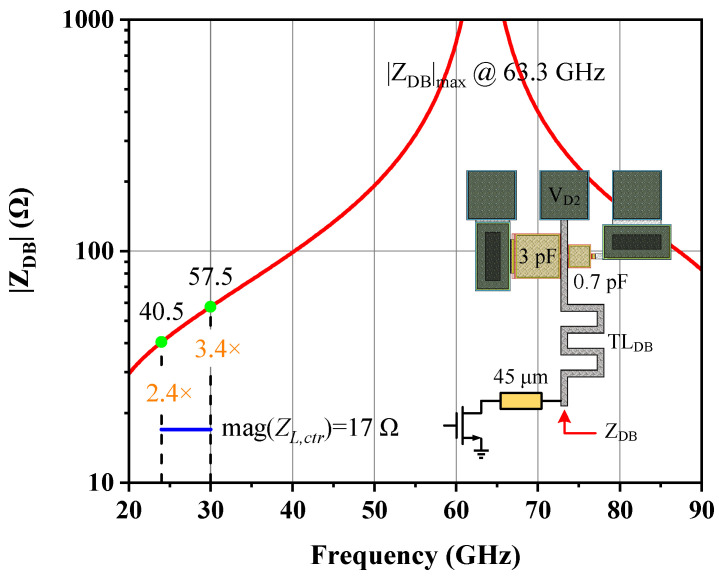
Drain bias network used in the output stage and its equivalent input impedance.

A good OMN is one that is concise and easy to implement, with adequate harmonic suppression outside the band. According to the ITR, FBW, and passband ripple of 4, 0.4, and 0.1, respectively, a fourth-order Chebyshev low-pass filter was adopted as the matching prototype, where the normalized *g* value of each element was determined by lookup tables in [9] and then scaled to the 50 system and 27 GHz center frequency *f*_0_ to obtain the preliminary inductance and capacitance. Afterward, parameters of the acquired real-to-real network were automatically adjusted in the order of random-before-gradient type with the help of an ADS optimizer, which finally transformed the intermediate impedance of 12 Ω to the desired *Z_L,ctr_* [13]. Following the rules described in the preceding subsection to translate an OMN into a mixed-element form, a short-circuit stub TL_DB_ was subsequently inserted as a drain bias branch, whose layout is displayed in Figure 8, along with the magnitude of its equivalent input impedance Z_DB_. The characterized open-circuit point lies at 63.3 GHz, somewhat higher than the second harmonic of the top fundamental frequency. Owing to the area limitation, the physical length of TL_DB_ cannot be increased aggressively and the current equivalent effect of close to λ/8 makes the |Z_DB_| provided in the target band only 2.4–3.4 times larger than the |*Z_L,ctr_*|, which is well below the 100 Ω magnitude. Therefore, the OMN must be further optimized to minimize the disturbance to the original frequency response after integrating the indispensable TL_DB_. It is worth noting that the bias tee was intentionally placed 45 μm away from the transistor’s drain terminal, leaving this physical connection as a decoupling spacer to lessen the influence of the bias trace on the cell, which can only be presented with an established vendor model so that these inevitable EM interferences are not reflected in the simulation process.

**Figure 9:**
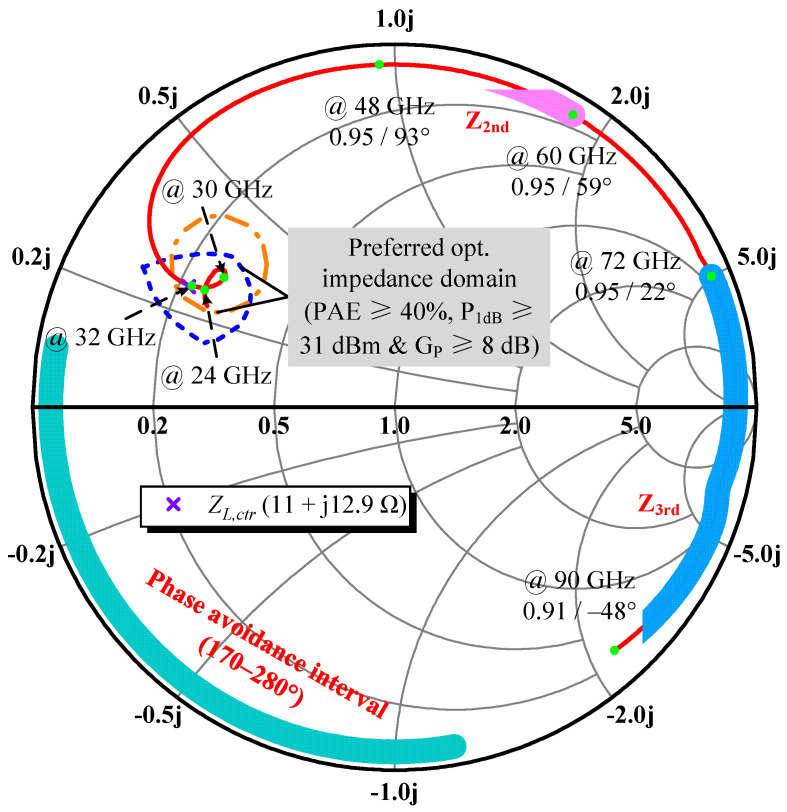
EM-simulated transformation feature (S_11_) of the OMN from 22 to 90 GHz.

Figure 9 shows how the response curve of the OMN rotates with the frequency inside the predefined optimal impedance domain and forms a knot signifying broadband matching, the junction of which corresponds to frequencies of 24 GHz and 31.5 GHz. Although the 22–32 GHz trajectory fails to sandwich *Z_L,ctr_* perfectly, it manages to fall into the narrow, preferred matching region as a whole, and the impedance at 32 GHz highly coincides with *Z_L,ctr_*, implying that the DA’s performance at the high-end fundamental is well secured and will not sharply deteriorate there. Furthermore, the second and third harmonic impedances exhibit relatively widespread scattering alongside the Smith chart’s margin, but neither are located in the aforementioned phase avoidance interval and therefore do not require extra harmonic manipulation.

**Figure 10:**
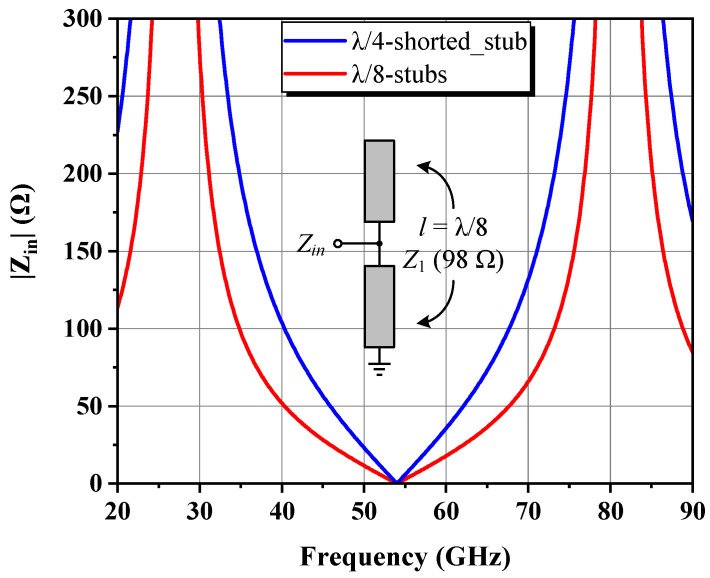
Impedance response of the ideal suggested even harmonic trap circuit designed at *f*_0_ compared with that of the shorted λ/4 transmission line.

The gate bias circuit of the output stage consists of open and shorted transmission lines with characteristic impedance *Z*_1_ (98 Ω for a single-layer metal microstrip that is 10 μm wide) and a length equal to λ/8 in parallel, as shown in Figure 10, whose input impedance *Z_in_* seen at the central node is expressed as Equation (17). Compared with the conventional quarter-wave short-circuit stub, the proposed structure not only serves the same function but also improves the layout flexibility, making full use of the free space on the top and bottom sides of the chip, and the extra multiplication factor of 0.5 helps to provide a better short-circuit termination state with extended bandwidth near the zeros, reducing even harmonic distortions from the front stage to be amplified together and mixed into the final product.

(17) $$Z_{\frac{\lambda }{8}-stubs}\left(f\right)=\frac{1}{2}jZ_{1}\tan\left(\frac{\pi }{2}\frac{f}{f_{fund}}\right)=\frac{1}{2}Z_{\frac{\lambda }{4}-shorted\_stub}$$

**Figure 11:**
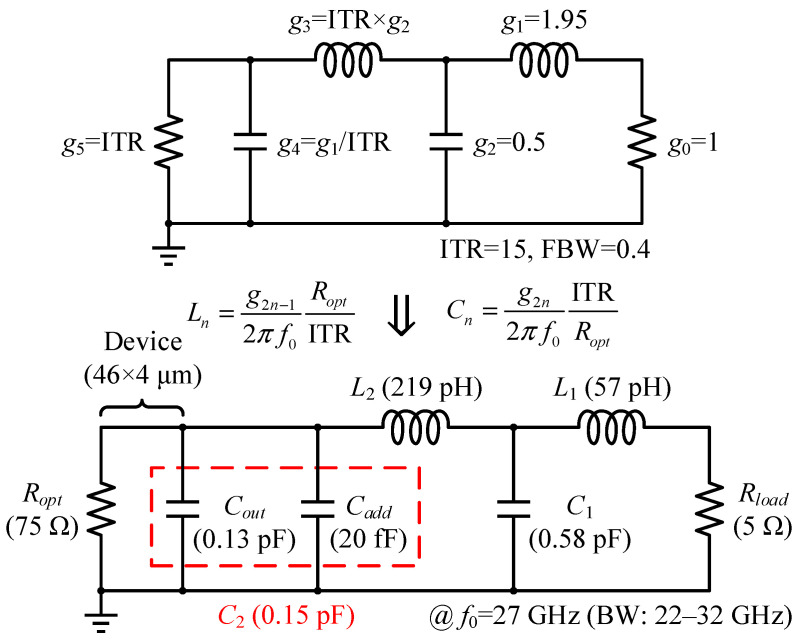
Conversion from a 0.1 dB equal-ripple low-pass filter prototype to a denormalized network.

After loading the EM model of the well-developed OMN to the output stage cell, source-pull simulations were conducted where a series 0.73 pF DC-block capacitor was placed. The real and imaginary parts of the resulting in-band *Z_S,opt_* vary in a small range, from 4.4 to 5.2 Ω and from 5.3 to –4.4 Ω, respectively, so the task of the interstage matching network (ISMN) can be simply specified as a real impedance transformation from 5 to 75 Ω. It would be more economical and advantageous for the broadband by participating the entire transistor’s intrinsic output parasitic contribution into matching, rather than canceling out *C_out_* through an inverse characteristic network or reoptimizing the finished real-to-real MN to compensate for it, which is important for the ISMN design that needs to cope with high ITR and simple requirements. Herein, a two-section Chebyshev impedance transformer centered at *f*_0_ was again adopted. Figure 11 illustrates a brief implementation flow of the ISMN for the lumped schematic phase, analogous to the OMN.

### 3.3. Input Impedance Model and the Synthesized Band-Pass IMN

**Figure 12:**
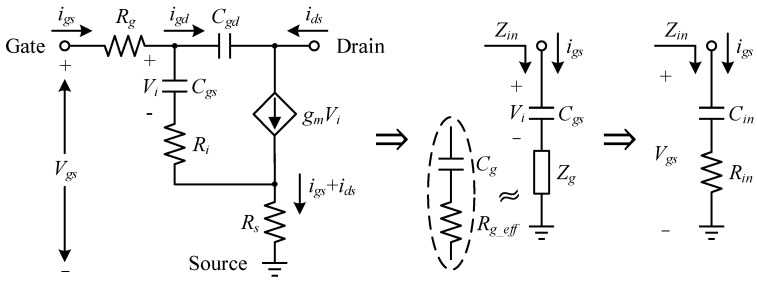
Equivalent circuit for the input of an HEMT in the common-source configuration and its simplified series RC model.

The input matching network (IMN) is responsible for providing a complex conjugate match at the driver stage cell’s input in order to optimize gain flatness and achieve a nice input VSWR. Contrary to the output case, *Z_in_* seen from the gate of a GaN HEMT has a series RC nature, as demonstrated below. Referring to Figure 12 [28], applying Kirchhoff’s laws to the small-signal equivalent circuit of the HEMT input, the gate impedance *Z_g_* can be derived.

(18) $$Z_{g}=\frac{V_{gs}-V_{i}}{i_{gs}}=R_{g}+R_{i}+R_{s}+\frac{i_{ds}}{i_{gs}}R_{s}-\frac{i_{gd}}{i_{gs}}R_{i}$$

Since *i_gd_* is small and there exists *i_gd_* ≪ *i_gs_*, neglecting it for the sake of simplicity, we have

(19) $$\frac{i_{ds}}{i_{gs}}=\frac{g_{m}V_{i}-i_{gd}}{V_{i}j\omega C_{gs}+i_{gd}}\approx \frac{g_{m}}{j\omega C_{gs}}$$

(20) $$Z_{g}\approx R_{g}+R_{i}+R_{s}+\frac{g_{m}R_{s}}{j\omega C_{gs}}=R_{g\_eff}+\frac{1}{j\omega C_{g}}$$

where

(21) $$C_{g}=\frac{C_{gs}}{g_{m}R_{s}}$$

It is evident that *Z_g_* can be regarded as a series connection of an effective gate resistor *R_g_eff_* formed by the sum of *R_g_*, *R_i_*, and *R_s_* plus a capacitor *C_g_*. Thus, *Z_in_* is expressed as

(22) $$Z_{in}=Z_{g}+\frac{1}{j\omega C_{gs}}=R_{g\_eff}+\frac{1}{j\omega }{\left(\frac{C_{gs}}{g_{m}R_{s}+1}\right)}^{-1}=R_{in}+\frac{1}{j\omega C_{in}}$$

**Figure 13:**
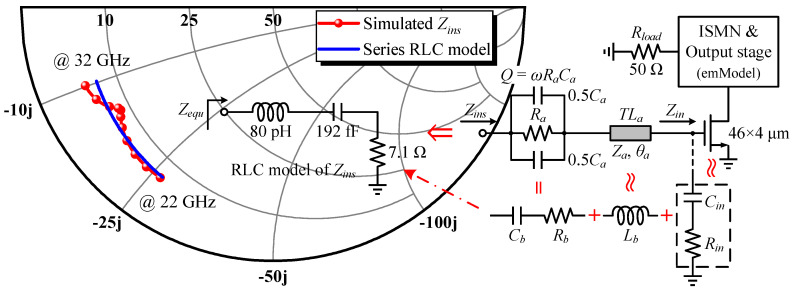
Input impedance characteristics of the stabilized 46 × 4 μm cell and the series RLC equivalent circuit.

**Figure 14:**
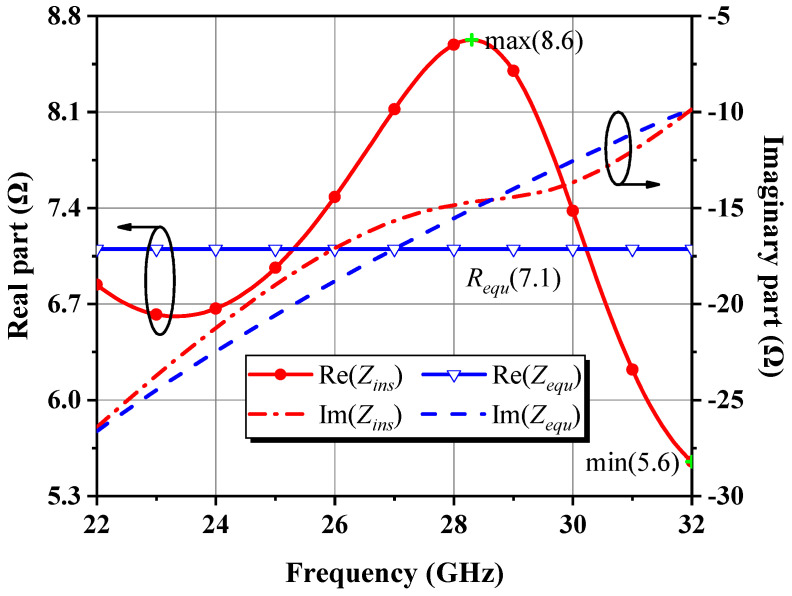
Comparison of the real and imaginary parts of *Z_ins_* and *Z_equ_*.

Therefore, the impedance *Z_ins_* seen at the new input node corresponds to a series RLC arrangement after the parallel *R_a_C_a_* stabilization network with a quality factor of *Q* and the connection line *TL_a_* are attached to the gate of the 46 × 4 μm cell loaded with all built-up post-stage circuits, expressed as Equation (23). It comprises a capacitor *C_b_* and a resistor *R_b_* resulting from the conversion of a parallel-to-series network, an inductor *L_b_* approximating *TL_a_*, and *Z_in_* simplified as *R_in_* in series with *C_in_*, as illustrated in Figure 13, where the parameters of the equivalent RLC model fitted from simulations, and the agreement between its characterized impedance *Z_equ_* and *Z_ins_* are also shown together. It is clear that the fit of the derived model is excellent at low frequencies in 22–32 GHz but declines to varying degrees as the frequency rises. Figure 14 reveals that this phenomenon is attributed to irregular changes in the real part of *Z_ins_*, whose peaks and valleys occur at 28.3 GHz and 32 GHz, respectively, and the resistance in the model circuit was taken as their arithmetic mean value of 7.1 Ω. Though the imaginary parts of *Z_ins_* and *Z_equ_* are closely matched within the 10 GHz bandwidth, there are no major undulating departures from each other, indicating that LC series resonance is the dominant form of *Z_ins_*’s reactance, despite its complex behavior. The simulation results confirm the RLC model suggested by the theoretical analysis and the acceptability of the selected circuit parameters.

(23) $$\begin{array}{ll} Z_{ins} & \approx \left(R_{b}+R_{in}\right)+j\omega L_{b}+\frac{1}{j\omega }\left(\frac{1}{C_{b}}+\frac{1}{C_{in}}\right)\\ & =\frac{R_{a}}{Q^{2}+1}+R_{g\_eff}+j\left[Z_{a}\sin\theta_{a}-\frac{Q^{2}}{\omega C_{a}\left(Q^{2}+1\right)}-\frac{g_{m}R_{s}+1}{\omega C_{gs}}\right] \end{array}$$

**Figure 15:**
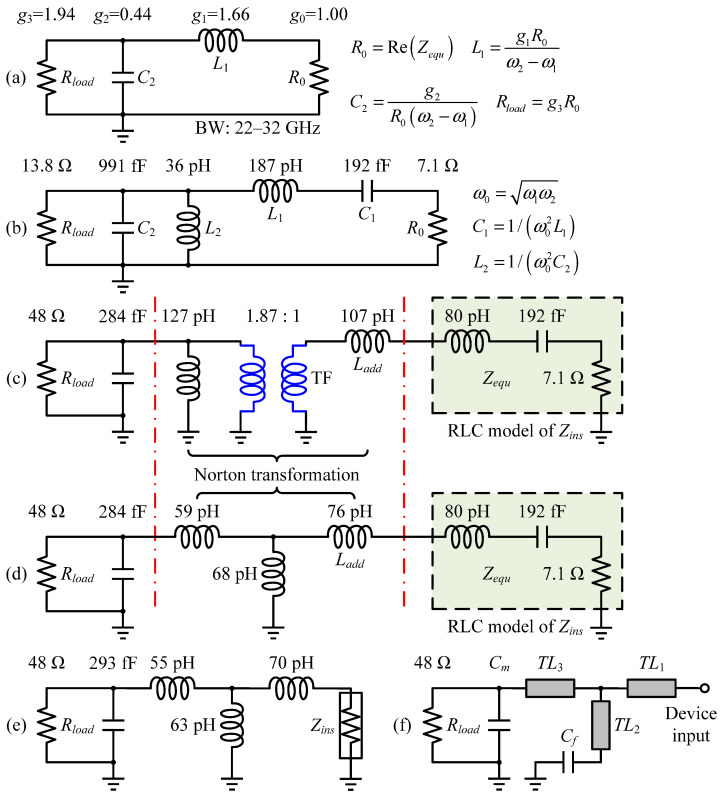
IMN design flow: (**a**) low-pass prototype after impedance and frequency scaling; (**b**) converted band-pass version; (**c**) upward impedance transformation of *R_load_* to 48 Ω; (**d**) Norton transformation to remove the ideal transformer; (**e**) network optimization for realistic *Z_ins_*; (**f**) corresponding ultimate IMN in the mixed-element format.

The dominant reactive constraint of the transistor input is a vital limiting factor for bandwidth extension, as is *C_out_*. Thus, the optimal realization form of the broadband IMN will be the band-pass structure to absorb the extracted series resonant components. As a rule of thumb, higher-order filters are able to broaden the bandwidth and achieve steeper stopband attenuation, but we should be aware that a rise in order requires more elements to be employed, more time for EM optimization procedures, and will not offer linear improvement [11]. Another aspect to emphasize is that the band-pass network costs twice the same-order low-pass counterpart. On balance, the IMN was built as a second-order band-pass filter using closed-form solutions via mathematical derivations [10,14,15], and the detailed design steps are given in Figure 15. Because the RLC model is not entirely equivalent to *Z_ins_*, the synthesized network must be fine-tuned for the actual frequency response before being converted into a mixed-element form, with the microstrip line *TL*_2_ serving as the gate bias path.

Given that the OMN is pivotal in deciding whether P_out_ and PAE objectives can be met, great efforts were undertaken early on and good outcomes were achieved. Hence, the joint-tuning phase solely entails adjusting the ISMN and the IMN. To enable a broadband gain response and input match, while maintaining favorable large-signal performance, concurrent small- and large-signal graded optimizations were carried out. The term “graded” refers to assigning more weight to optimization goals at the upper frequencies, which are an area of concern, so that a better fit gets produced sooner than with equal-weight settings.

### 3.4. Loss Analysis of the MN and Stability of DA

**Figure 16:**
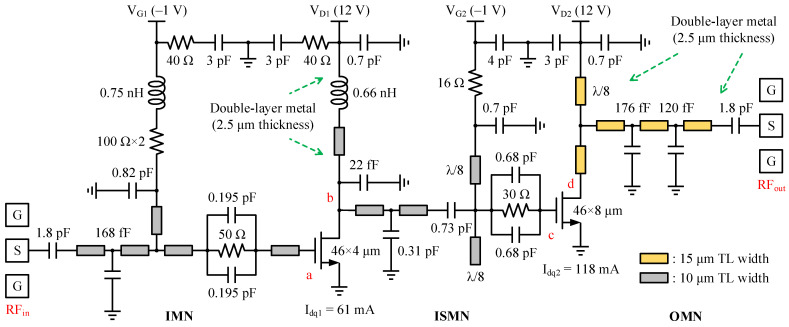
Schematic diagram of the two-stage DA. P_out_ is simulated at nodes a–d in Figure 17.

**Figure 17:**
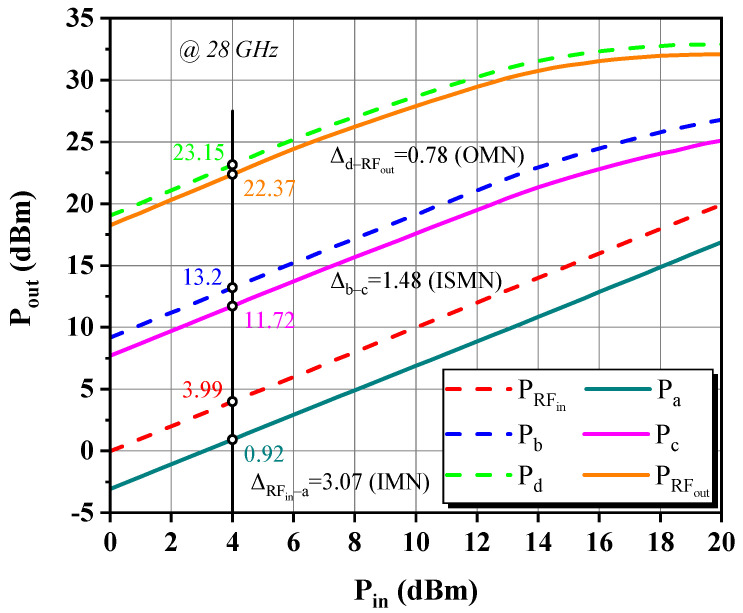
Simulated P_out_ at various nodes of the DA at 28 GHz.

**Figure 18:**
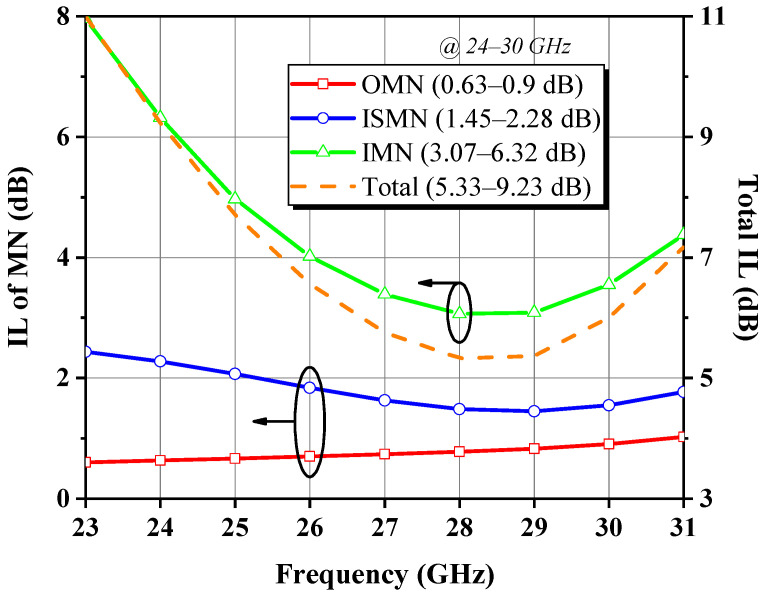
IL of each MN and all MNs as a function of frequency.

Figure 16 depicts the entire circuit schematic, where the gate and drain feeds for each stage are provided independently, isolated from one another to eliminate interference and thus improve stability. To quantify the insertion loss (IL) associated with the MN, i.e., to calculate the difference between the power entering and leaving the MN as described in Equation (24), the DA was injected with a fixed low-power stimulus *P_in_* away from the 1 dB gain compression point. Based on Figure 17 for an intuitive illustration, the six power sweep curves corresponding to various nodes indicated by the red labels in Figure 16 are linearly increasing in the interval of *P_in_* less than 10 dBm, and the power difference between nodes in such class-A operation is nearly constant. Therefore, we read all node power with an arbitrary *P_in_* of 4 dBm and obtained ILs of 0.78, 1.48, and 3.07 dB for the OMN, ISMN, and IMN at 28 GHz, respectively. When the RF output power is progressively saturated, the curve P_c_ displays a quasi-proportional growth trend mildly deviating from linearity, which denotes that the front stage neither works in the deep back-off region of low efficiency nor demonstrates insufficient driving power, avoiding unnecessary DC consumption and distortion generation. As a consequence, the bias Q-point and the staging ratio of 1:2 settings were verified to be appropriate to some extent. Likewise, the overall frequency responses shown in Figure 18 can be obtained. To achieve the highest possible PAE in the context of compactness, the OMN was implemented in a simple structure and used high characteristic impedance microstrip lines with a double-layer metal, whose maximum IL does not exceed 0.9 dB over 24–30 GHz. In contrast, the IL trend of the ISMN and the IMN compensates well for the device’s negative gain roll-off slope, with the IMN being the main contributor, which reveals the reason for its large low-frequency loss. The total IL of three MNs is regulated within 9.23 dB in the designed band and exhibits a trend of rapidly decreasing with increasing frequency and then leveling off and steadily growing, with the valley at 28 GHz, corresponding to a minimum value of 5.33 dB.

(24) $$\mathrm{IL}=P_{in\_MN}-P_{out\_MN}$$

**Figure 19:**
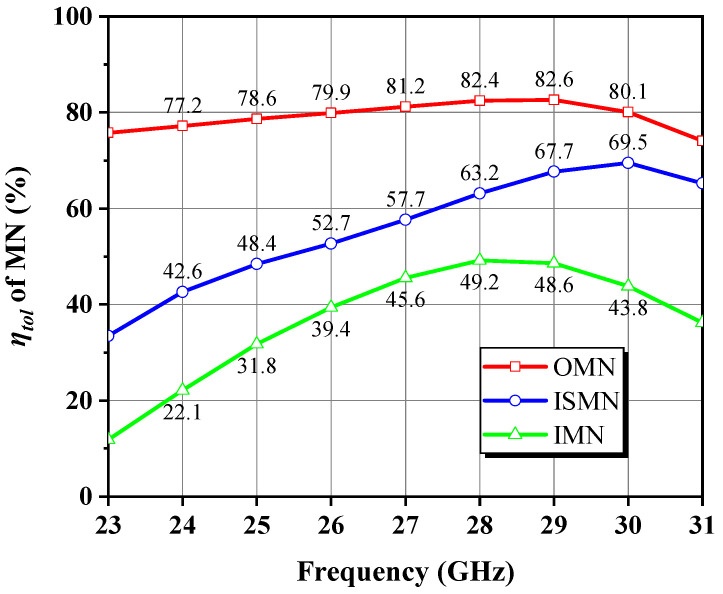
*η_tol_* of each MN across 23–31 GHz.

Apart from the inherent dissipation loss of the MN, another is the mismatch loss, which together ensures the gain flatness of the DA. The total efficiency *η_tol_* of a lossy MN is defined as the ratio of the delivered power *P_L_* (i.e., *P_out_MN_*) absorbed by load to the available power *P_avs_* from the source *V_g_*, expressed as

(25) $$\eta_{tol}=\frac{P_{L}}{P_{avs}}=\frac{P_{in\_MN}}{P_{avs}}\times \frac{P_{out\_MN}}{P_{in\_MN}}=\eta_{match}\times \eta_{loss}$$

where *η_loss_* is the MN’s transmission efficiency, which equals 1 in the absence of the MN. The matching efficiency *η_match_* can be further derived as Equation (26), which equals 1 in the case of conjugate matching, where *Z_S_* = *R_S_* + j*X_S_* is the source impedance and *Z_in_* = *R_in_* + j*X_in_* is the input impedance of the MN with a load attached. Table 3 details the simulated *η_match_* and *η_loss_* for all MNs in the range of 23–31 GHz, and their product *η_tol_* is plotted in Figure 19. These findings validate the effectiveness of the filter synthesis theory in creating broadband MNs and explicitly reflect the design focus of each MN. The OMN scores well among various efficiency indicators, where the in-band *η_tol_* surpasses 77.2% and fluctuates no more than 5.4%, ensuring a smooth and low-loss power transfer. As for the ISMN, *η_match_* shows a more prominent trend of increasing with frequency than *η_loss_*, with the difference between the highest and lowest fundamental frequencies of *η_match_* being up to 27.2%, which represents the broadband strategy implemented by the ISMN to better match at the high-frequency side and introduce a proper low-frequency mismatch to cope with the demanded driving power of the output stage growing with frequency. In addition, *η_loss_* remains above 59.2%, so the adverse impact of the associated IL on gain and PAE is still tolerable. Thanks to the applied band-pass filter structure, the IMN manifests superior and nearly uniform *η_match_*. Nonetheless, we intentionally sacrifice *η_loss_* to attain an eventual broadband gain response with enhanced stability in return for losing more than half of the transmitted signal energy, which seldom compromises large-signal performance. Overall, they exhibit a gradient decreasing *η_tol_* from the OMN to the IMN, with peaks lying in the upper-frequency portion (28–30 GHz).

(26) $$\eta_{match}=\frac{P_{in\_MN}}{P_{avs}}=\frac{{\left|\frac{V_{g}}{\left(R_{S}+R_{in}\right)+j\left(X_{S}+X_{in}\right)}\right|}^{2}R_{in}}{\frac{{\left|V_{g}\right|}^{2}}{4R_{S}}}=\frac{4R_{in}R_{S}}{{\left|Z_{in}+Z_{S}\right|}^{2}}$$

**Figure 20:**
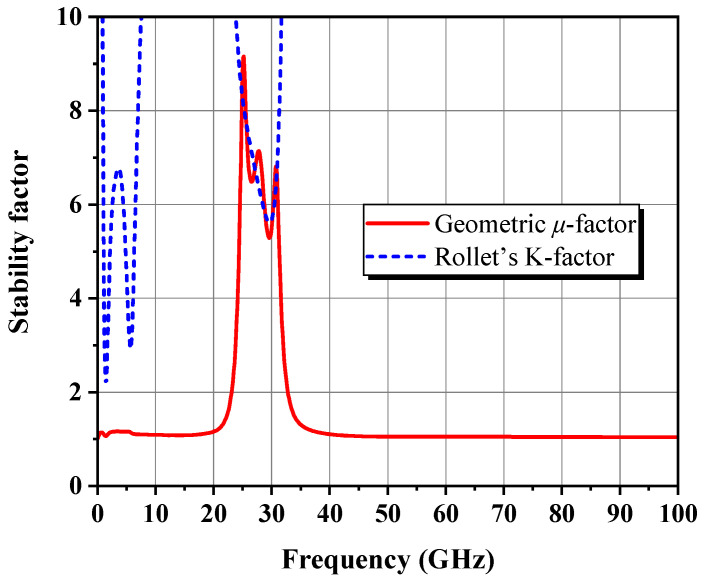
Stability factors of the final DA.

Before tape-out, it is mandatory to carefully check the overall stability of the DA. To ensure that the DA is unconditionally stable under any possible operating conditions, countermeasures were incorporated into the design by (1) connecting a parallel RC network in front of the two transistors, (2) inserting a resistor in both gate bias paths, (3) paralleling a series combination of a 3 pF bypass capacitor and a 40 Ω resistor to the gate and drain supply lines of the driver stage, and (4) in conjunction with the resistive losses of all MNs. Figure 20 proves that the simulated stability factors *μ* and K are greater than unity over DC–100 GHz.

## 4. Probed Measurement Results

**Figure 21:**
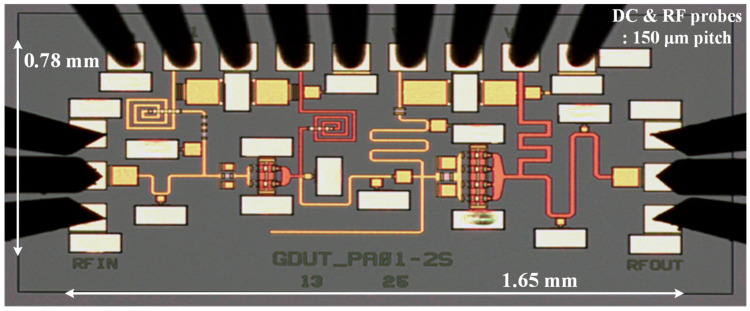
Micrograph of the 24–30 GHz GaN MMIC DA together with DC and GSG-type RF probe card tips.

Instead of assembling the bare die into a fixture to avoid bonding inductive interconnected gold wires to the RF pads and causing uncertainty in the original impedance matching, we conducted on-wafer testing of the fabricated MMIC DA using ground–signal–ground (GSG) microwave probes and dedicated DC probe cards for proper DC decoupling with a manually-controlled probe station. Figure 21 shows a close-up micrograph where tapers were added that fit with the device drain pin width to reduce step junction discontinuities. However, the thermal contact between the backside of the chip and the heat sink surface will be poor and lead to inferior heat dissipation [22], which may provoke thermal degradation or even burnout of the transistor. To prevent overheating, a 12 V pulsed supply with a 5% duty cycle (200 μs pulse width and 4 ms period) was applied. First, performing necessary but quick partial stability inspections, the gate bias voltage was slowly raised from the threshold of –1.5 V to bring the quiescent current draw of the DA to 179 mA, in line with the simulation environment in the absence and presence of weak sinusoidal excitation, with no oscillations monitored across the full spectrum during both processes. Afterward, a series of measurements were carried out at room temperature using the N5183A MXG analog signal generator, N5245A PNA-X network analyzer, and N9021B MXA signal analyzer from Keysight (Santa Rosa, CA, USA).

### 4.1. Small-Signal Characterization

**Figure 22:**
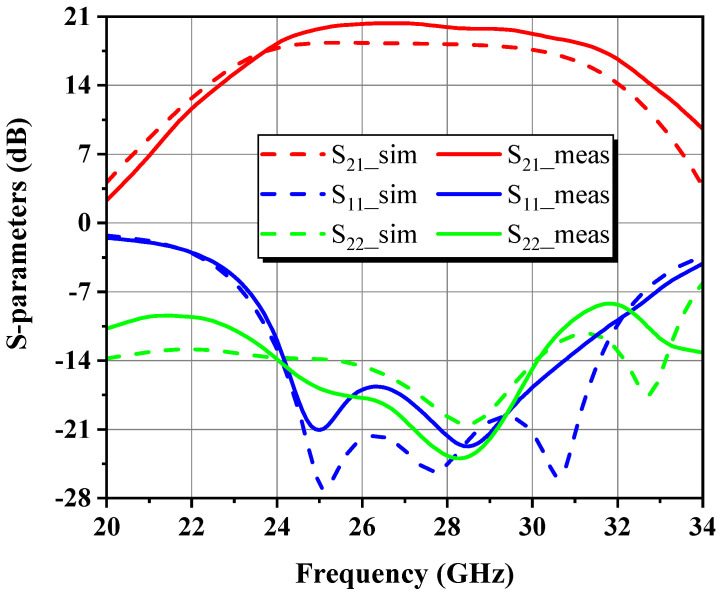
S-parameters of the proposed DA.

Figure 22 exhibits that S_21_ ranges between 18.3 and 20.3 dB within the 24–30 GHz operating bandwidth, which is higher than predicted in the CW mode from 24 GHz onward, with an average in-band excess of about 1.7 dB. Simulated S_11_ gives a distinct band-pass Chebyshev equal-ripple response, whereas S_22_ is relatively poor, as described from the conceptual standpoint that the output stage is power matching via load-pull instead of small-signal conjugate matching, so the S_22_ performance is sacrificed at some expense in exchange for enhanced P_out_. Their respective in-band measured values stay below –12 dB and –13.9 dB, suggesting that the input/output ports are well matched.

### 4.2. Large-Signal Characterization

**Figure 23:**
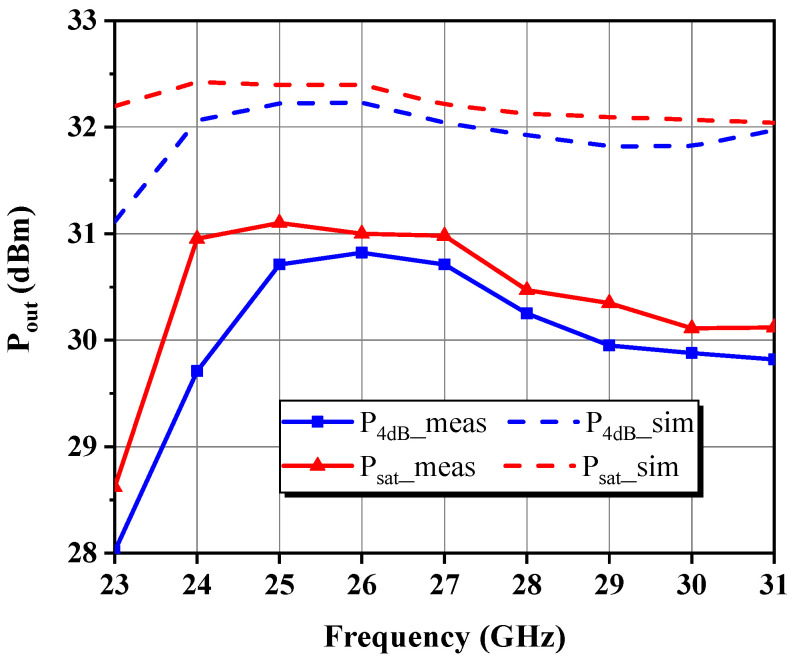
Measured and simulated P_out_ at 4 dB gain compression and in the saturation state.

**Figure 24:**
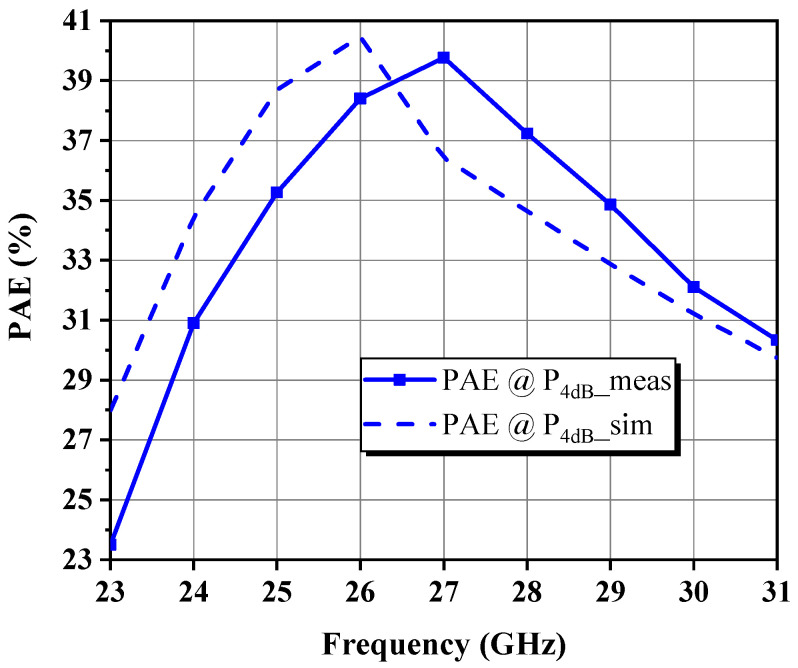
Measured and simulated PAE at P_4dB_ versus frequency.

**Figure 25:**
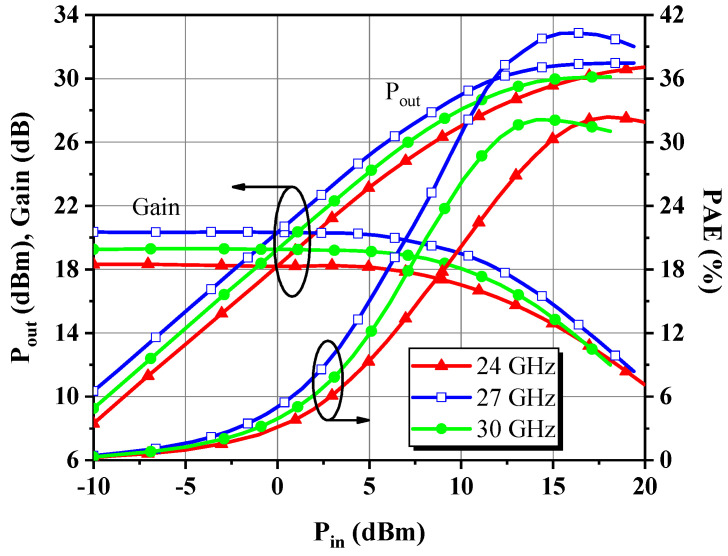
Measured P_out_*,* PAE, and gain behaviors with respect to P_in_ at three equally spaced frequencies across the objective band.

A close examination of Figure 23 and Figure 24 reveals that the collected P_4dB_ and P_sat_ over the band of interest are 29.7–30.8 dBm and 30.1–31.1 dBm, respectively, following almost the same trend as expected, with only an overall decline of about 1.7 dB and 1.5 dB from their simulated counterparts. The PAE at P_4dB_ exceeds 30.9% in the band, with a peak of 39.8% at *f*_0_, and the curve is displaced by 1 GHz to higher frequencies compared to the simulation. Figure 25 depicts power sweep curves at low, medium, and high fundamentals, which show that the DA characterizes the anticipated soft compression behavior, generally reaching the critical saturation state in the vicinity of P_6dB_, but more boost power is required for the low-frequency case. This might be because of nonlinear trapping effects, which are more pronounced in GaN-on-Si than GaN-on-SiC, as the former has a higher defect density. In addition, the highest PAE occurs at about 2 dB back from the saturation point and total current consumption grows to 241–283 mA when the DA is driven into the 8 dB gain compression state. No case of instability was detected throughout the tests. Therefore, the applied stability measures seem to be sufficient.

The measured and simulated outcomes agree well in terms of trends, but they differ somewhat in data values and also partly show slight frequency deviations, which are mainly attributed to: the accuracy of the transistor modeling; inevitable and stochastic manufacturing errors, especially in MIM capacitors; channel temperature variations due to self-heating from gate finger dissipation; the calibration status of the entire test system; and the contact quality between the probes and RF pads. Note that the nonlinear scalable model of the device provided by the foundry was established on the basis of the measurement of typical test kits, and the ones with specific sizes used here were extrapolated through mathematical fitting from several validated samples. Therefore, they may not accomplish the same high accuracy as typical cells. Table 4 summarizes the experimental results in comparison to previously reported PAs with similar frequency bands.

## 5. Conclusions

This paper demonstrates a fully integrated 1-watt GaN MMIC DA for the 24–30 GHz band. The optimal impedance domain with a moderate range was delineated through simplified load-pull simulations according to reasonably preset goals. The reference center impedance *Z_L,ctr_* was calculated by weighted averaging, with an emphasis on high-frequency PAE. As per the proposed target space consisting of an area and a single point instead of discrete compromise matching points at different fundamental frequencies or arcs formed by theoretical impedance sets, as in class-J amplifiers, the implementation process avoids addressing the tricky impedance-tracking problem and relieves the pressure of broadband design. The low-loss OMN without harmonic control circuitry was thereafter developed applying the Chebyshev filter synthesis theory and paired with CAD post-optimization to ensure that the transformed in-band impedance trajectory nestles inside the preferred region, which can better solve the contradiction between multiple metrics under a broadband. With the same fourth-order low-pass ladder based on a Chebyshev response, the ISMN differs from the OMN in that it exploits the transistor’s *C_out_* de-embedded from load-pull contours to complete the compact broadband matching at an ITR as high as 15. For the series RLC model established by theoretical analysis and simulation, equivalent to the stabilized cell’s input impedance, the IMN was realized as a band-pass filter using closed-form solutions to absorb the resonant input parasitics, achieving a return loss of 12 dB minimum in the 6 GHz bandwidth. The step-by-step circuit construction strategy rather than direct modular assembly allows the remaining MNs to be designed on the basis of frequency responses from a progressively built and refined EM model, which lessens the risk of unmitigated performance deviations or laborious adjustments during the joint-tuning phase, thus increasing the design efficiency and success rate. Moreover, this is greatly beneficial for amplifiers with larger-scale architectures, such as N-way combining and differential, as the reduction in simulation time is more significant than with conventional ways. The answers provided in this work in tackling broadband problems were outlined in terms of analyzing the losses of each MN, and experimental results validate the proposed design scheme. The realized two-stage DA has a linear gain of 19.3 dB on average, with a less than 1 dB fluctuation in the operating bandwidth and a state-of-the-art PAE of up to 39.8%, with tight integration and a small size of 1.29 mm^2^, contributing to lower fabrication and production costs, all of which prove the suitability of the presented design for massive commercial 5G mmW applications.

## Figures and Tables

**Table 1:** Simulation comparison between the conventional and simplified fundamental load-pull procedures for the stabilized 46 × 8 μm cell with the purpose of achieving the maximum PAE.

Freq. (GHz)	*Z_L__,opt_* (Ω)	*Z_S,opt_* (Ω)	*Z_S,fund_* (Ω)	*PAE_conv_*_._ (%)	*PAE_simpl_*_._ (%)
24	13.6 + j16.9	4.9 + j10.5	4.9 + j9.8	54.2	53.9
28	9.9 + j14.5	4.6 + j7.4	4.7 + j7.8	50.8	50.9
32	9.2 + j11.8	5.3 + j6.2	4.9 + j6.1	47.4	47.5

**Table 2:** Contour peaks and weighted averages at discrete frequencies.

Freq. (GHz)	*P*_1_ (Ω)	*P*_2_(Ω)	*P_i_* (Ω)	*W_i_* (%)	*Z_L,ctr_* (Ω)
24	13.6 + j16.9	15.9 + j12.1	14.4 + j15.3	20	11 + j12.9
28	9.9 + j14.5	11.5 + j10.6	10.4 + j13.2	50	
32	9.2 + j11.8	10.4 + j8.5	9.6 + j10.7	30	

**Table 3:** *η_match_* and *η_loss_* of each MN across 23–31 GHz.

Freq. (GHz)	OMN		ISMN		IMN	
	*η_match_* (%)	*η_loss_* (%)	*η_match_* (%)	*η_loss_* (%)	*η_match_* (%)	*η_loss_* (%)
23	87	87.1	58.6	57.1	75.3	15.7
24	89.3	86.4	72	59.2	94.8	23.3
25	91.7	85.8	78	62.1	99.8	31.8
26	93.9	85.1	80.5	65.5	99.4	39.6
27	96.2	84.4	83.9	68.7	99.5	45.8
28	98.6	83.6	88.9	71.1	99.6	49.4
29	100	82.6	94.5	71.6	99	49.1
30	98.6	81.2	99.2	70	99.3	44.1
31	93.8	79	98.1	66.5	99.3	36.5

**Table 4:** Performance summary and comparison with contemporary works.

Ref.	[2]	[17]	[18]	[19]	[20]	TGA2594 [29]	This Work
Process	0.15 μm GaAs	0.1 μm GaN/Si	0.1 μm GaN/SiC	0.15 μm GaN/SiC	0.1 μm GaN/Si	0.15 μm GaN/SiC	0.1 μm GaN/Si
V_D_ (V)	5	12	15	20	12	20	12
Meas. mode	CW	Pulsed	Pulsed	CW	Pulsed	CW	Pulsed
Freq. (GHz) (FBW)	25–29 (14.8%)	22–27 (20.4%)	27–34 (23%)	32–38 (17.1%)	24–30 (22.2%)	27–31 (13.8%)	24–30 (22.2%)
Gain (dB)	22.7 ± 0.7	24 ± 0.5	20.5 ± 1.5	17 ± 0.5	17.9 ± 1.5	23.6 ± 1.9	19.3 ± 1
P_out_ (dBm)	26 ± 0.3	31 ± 0.7	38.7 ± 0.4	36.7 ± 0.5	39.9 ± 1	37 ± 0.4	30.6 ± 0.5
PAE (%)	27.2–32.5 ^a^	30.5–36.9 ^b^	24.5–30.5 ^a^	25–34 ^c^	24–37 ^b^	26.5–30.3 ^c^	30.9–39.8 ^a^
Size (mm^2^)	2 × 1.3	1.8 × 0.87	4.5 × 3.5	2.22 × 1.6	3.7 × 3.2	3.24 × 1.74	1.65 × 0.78

^a^ PAE @ specific gain compression point. ^b^ PAE @ saturated output power. ^c^ PAE @ specific input drive.

## Data Availability

Not applicable.
